# Oridonin represses epithelial-mesenchymal transition and angiogenesis of thyroid cancer via downregulating JAK2/STAT3 signaling

**DOI:** 10.7150/ijms.70733

**Published:** 2022-05-27

**Authors:** Wei Liu, Xindi Wang, Le Wang, Yu Mei, Yanning Yun, Xiaobao Yao, Qian Chen, Jinsong Zhou, Bo Kou

**Affiliations:** 1 Department of Vascular Surgery, The First Affiliated Hospital of Xi'an Jiaotong University, Xi'an, Shaanxi 710061, China; 2 Department of Clinical Medicine, Medical School of Xian Jiaotong University, Xi'an, Shaanxi 710061, China; 3 Department of Otorhinolaryngology-Head&Neck Surgery, The First Affiliated Hospital of Xi'an Jiaotong University, Xi'an, Shaanxi 710061, China; 4 Department of Otorhinolaryngology-Head&Neck Surgery, The First Affiliated Hospital of Xi'an Medical University, Xi'an, Shaanxi 710061, China; 5 Department of Human Anatomy, Histology and Embryology, School of Basic Medical Sciences, Xi'an Jiaotong University Health Science Center, Xi'an, Shaanxi 710061, China; 6 Key Laboratory of Environment and Genes Related to Diseases, Xi'an Jiaotong University, Ministry of Education of China, Xi'an, Shaanxi 710061, China

**Keywords:** oridonin, thyroid cancer, EMT, angiogenesis, JAK2, STAT3

## Abstract

Oridonin, a bioactive diterpenoid isolated from *Rabdosia rubescens*, has been reported to exert anticancer activity in various cancers. However, the molecular mechanism of oridonin in thyroid cancer has not yet been elucidated. In the present study, oridonin was found to significantly inhibit migration and invasion of thyroid cancer TPC-1 and BCPAP cells, as evidenced by wound healing assay, transwell migration assay and Matrigel invasion assay. In addition, oridonin could partially impede epithelial-mesenchymal transition by upregulating E-Cadherin expression and downregulating N-Cadherin and vimentin expressions in a concentration-dependent manner. Accumulating evidence indicated that JAK2 (Janus kinase-2)/STAT3 (Signal Transducer and Activator of Transcription 3) signaling pathway was associated with epithelial-mesenchymal transition. As expected, the protein levels of phosphorylated-JAK2 and phosphorylated-STAT3 were dramatically reduced upon oridonin treatment in thyroid cancer TPC-1 and BCPAP cells. Subsequently, the findings revealed that JAK2 overexpression could weaken the anti-metastatic effect and partially attenuate MET (mesenchymal-to-epithelial transition) by oridonin, while AG490, a JAK2 antagonist, enhanced the above process in thyroid cancer cells. The subsequent results showed that oridonin inhibited angiogenesis and VEGFA expression in thyroid cancer cells by tube formation assay, western blot and ELISA assay. Meanwhile, AG490 could further attenuate oridonin-treated VEGFA protein level. In addition, the *in vivo* results further confirmed that oridonin inhibited tumorigenicity in thyroid cancer xenograft. In conclusion, the results demonstrated that oridonin repressed metastatic phenotype, angiogenesis and modulated EMT (epithelial-mesenchymal transition) of thyroid cancer cells via the inactivation of JAK2/STAT3 signaling pathway, suggesting that JAK2 may be a novel therapeutic target of oridonin against thyroid cancer.

## Introduction

Thyroid cancer is the most common malignancy in endocrine system, with an increasing morbidity and mortality 1. Papillary thyroid cancer (PTC) is the most common type of thyroid cancer, accounting for nearly 80% of all thyroid cancers 2. Benefits from the improvement of diagnosis and treatment, most patients with PTC have a favorable prognosis. Nevertheless, there is still a significant proportion of recurrence and metastasis after regular treatment in PTC patients. Moreover, the molecular mechanisms of thyroid cancer recurrence and metastasis have not yet been elucidated. Therefore, it is of great necessity to find out the potential therapeutic agents against PTC and to explore the mechanism of cancer cell invasion and metastasis.

Oridonin, a bioactive diterpenoid isolated from *Rabdosia rubescens*, has been reported to exert broad pharmacological and physiological effects in the past decades, such as anti-inflammation and anti-tumor effects 3-5. In addition, studies revealed that oridonin possessed remarkable suppressive activity against ovarian cancer, lung cancer and gastric cancer 6-8. Studies showed that many signaling pathways were involved in the process, including ROS (Reactive Oxygen Species)/JNK (Jun N-terminal Kinase)/c-JUN axis, PI3K (Phosphatidylinositol 3 Kinase)/AKT (Protein Kinase B) signaling and Notch signaling 9-11. For thyroid cancer, the impact of oridonin has not yet been reported.

Herein, we aimed to explore the inhibitory effects of oridonin on migration, invasion, epithelial-mesenchymal transition and angiogenesis in thyroid cancer TPC-1 and BCPAP cell lines. The regulatory mechanism about oridonin represses metastatic phenotype and angiogenesis would be also confirmed.

## Materials and methods

### Reagents and cell culture

Oridonin (C_20_H_28_O_6_) was obtained from Sigma-Aldrich (St. Louis, MO, USA) and dissolved in dimethyl sulfoxide (DMSO). Stock solutions were stored at -20 °C. Antibodies against phosphorylated-Janus Kinase-2 (p-JAK2)(Tyr1007/1008, #3776, 1:1000), JAK2 (#3230, 1:1000), phosphorylated-Signal Transducer and Activator of Transcription 3 (p-STAT3)(Tyr705, #9145, 1:1000), STAT3 (#30835, 1:1000), E-cadherin (#14472, 1:1000), N-cadherin (#13116, 1:1000), Vimentin (#5741, 1:1000) and β-actin (#4970, 1:1000) were obtained from Cell Signaling Technology, Inc (Beverly, MA, USA). Antibody against VEGFA (#ab46154, 1:1000) was purchased from Abcam, Inc (Cambridge, Britain). The ELISA kit was gained from RayBiotech Inc (Norcross, GA, USA). 3-(4,5-dimethylthiazol-2-yl)-2,5-diphenyltetrazolium bromide (MTT) was acquired from Sigma Chemical Co. (St. Louis, MO, USA).

Human thyroid cancer cell lines TPC-1, BCPAP, thyroid cell line Nthy-ori 3-1 and human umbilical vein endothelial cell line HUVEC were obtained from the American Type Culture Collection (Manassas, VA, USA). The cell lines were cultured in 1640 medium, which contains with 10% fetal bovine serum (Gibco, Grand Island, NY, USA) and seeded at 37 °C in a humidified incubator with 5% CO_2_.

### Cell proliferation assay

The proliferation viability was determined by a modified MTT assay 12. In brief, 1.0 × 10^4^ TPC-1 and BCPAP cells were seeded into 96-well plates at 200 μl per well. After treatment with DMSO or the increasing concentrations of oridonin (1, 2.5, 5, 10, 15, 20, 25 μM) for 24 h, 20 μl of MTT dye solution (5.0 mg ml^-1^) was added to each well with 180 μl medium. After incubation in the cell incubator for another 4 h, cells were lysed with dimethyl sulfoxide (DMSO) to dissolve the formazan crystals. Then the optical density (OD) was measured at 490 nm wavelength on microplate reader (Bio-Rad, Hercules, CA, USA). The growth inhibitory rate was calculated as: [(OD 490_control cells_-OD 490_treated cells_)/ OD 490_control cells_] ×100. Three independent experiments were performed.

### Wound healing assay

Thyroid cancer TPC-1 and BCPAP cells were plated onto six-well plates until 90 % confluence, then scratch wounds were created across the monolayer with the tip of a 200-µl pipette. After incubation in a serum-free medium with oridonin treatment, five fields (100 ×) were randomly chosen from each scratch wound and images were captured by microscope to evaluate the migratory capacity. Three independent experiments were performed.

### Transwell migration assay

For the transwell migration assay, it was performed via chambers (Millipore, Billerica, MA, USA) with an 8-μm pore size. 4 × 10^4^ TPC-1 or BCPAP cells with 200 μl serum-free medium were treated as above and plated onto the upper chamber, while 800 μl of medium with 10 % fetal calf serum were then plated onto the lower chamber in 96-well plate. After certain incubation at 37 °C, the non-migrating cells on the top chambers were completely removed with a cotton swab. The cells that migrated to the lower surface of the filter were then fixed with 4 % paraformaldehyde and stained by 0.1% crystal violet (Beyotime, Shanghai, China). Cells in five random fields were then counted and visualized using a microscope at 100 × magnification. Three independent experiments were performed.

### Matrigel invasion assay

The impact of oridonin on the invasion of thyroid cancer TPC-1 and BCPAP cells were assessed by matrigel invasion assay with a Millicell chamber (Millipore, Billerica, MA, USA). The membrane with 8-μm pore size in the upper chamber was pre-coated with 50 μl Matrigel (Matrigel: serum-free medium 1:5). The rest of the steps is similar to the Transwell migration assay. Three independent experiments were performed.

### Quantitative real-time PCR assay

TPC-1 and BCPAP cells were pre-treated with different concentrations of oridonin and the total RNA were extracted using TRIzol reagent (Invitrogen, Carlsbad, CA, USA) following the manufacturer's instructions. Then the RNA was reversely transcribed to complementary DNA (cDNA) using a PrimerScript RT reagent Kit (Takara, Dalian, China). Subsequently, the quantitative real-time PCR (Polymerase Chain Reaction) was performed using the SYBR Green Master Mix. The sequences of primers for PCR amplification were forward 5'-CGAGAGCTACACGTTCACGG-3' and reverse 5'-GGGTGTCGAGGGAAAAATAGG-3' for E-Cadherin (119 bp); forward 5'-TCAGGCGTCTGTAGAGGCTT-3' and reverse 5'-ATGCACATCCTTCGATAAGACTG-3' for N-Cadherin (94 bp); forward 5'-GACGCCATCAACACCGAGTT-3' and reverse 5'-CTTTGTCGTTGGTTAGCTGGT-3' for Vimentin (238 bp); forward 5'-CATGTACGTTGCTATCCAGGC-3' and reverse 5'-CTCCTTAATGTCACGCACGAT-3' for β-actin (250 bp). The n-fold change in mRNA expression was calculated with the 2^-ΔΔCt^ method. Three independent experiments were performed.

### Western blotting

Briefly, thyroid cancer TPC-1 and BCPAP cells were collected after certain treatment, and the proteins were extracted using protein lysis buffer. After centrifugation and denaturation, the extracts (about 30-60 µg) were subjected to SDS (Sodium Dodecyl Sulfate)-polyacrylamide gel electrophoresis (10 % or 15 %) and transferred to polyvinylidene fluoride membranes (Millipore, Bedford, MA, USA). Membranes were then probed with corresponding antibodies against E-cadherin, N-cadherin, Vimentin, p-JAK2, JAK2, p-STAT3, STAT3 and β-actin overnight at 4 °C, respectively. Subsequently, the protein bands were washed with TBST and incubated with horseradish peroxidase (HRP)-conjugated IgG antibody at room temperature. Ultimately, the protein bands were visualized using ECL (Electrochemiluminescence) Substrate and exposed to X-ray film.

### Plasmid transfection

JAK2 cDNA was cloned into pcDNA3.1 vector. After thyroid cancer TPC-1 or BCPAP cells reached 80 % confluency for plasmid transfection, the cells were transiently transfected with X-treme GENE HP DNA Transfection Reagent (Roche, Germany) for certain time according to the manufacturer's instructions, and prepared for the next experiments.

### Conditional medium collection and tube formation assay

Thyroid cancer TPC-1 and BCPAP cells were seeded into the 6-well plates. After adherence, cells were washed with serum-free medium (SFM) twice. Then, cells were exposed to different treatments with SFM for 24 hours. Subsequently, the supernatant was collected and centrifuged to gain the conditional medium (CM).

For tube formation assay, HUVEC cells were re-suspended with CM and seeded into 24-well plates, which were pre-coated with 200 μl Matrigel (Matrigel: serum-free medium=1:5). The tube was visualized and counted 4-6 hours later using a microscope at 100 × magnification.

### ELISA assay

After collecting the conditional medium (CM), the concentration of VEGFA in CM was detected by the ELISA kit according to manufacturer's instructions. Three independent experiments were performed.

### Xenograft tumor model

Ten four-week-old male BALB/C nude mice were purchased from and maintained in the Animal Care and Use Committee of Xi'an Jiaotong University, Xi'an, China. All the animal experiments were approved by the Ethics Committee of Animal care and Use of Xi'an Jiaotong University. The nude mice were randomly and equally divided into two groups, and subcutaneously injected with 100 μl serum-free medium containing 5×10^6^ TPC-1 cells into right flank of mice. After the tumor volumes of the xenografts reached approximately 90 mm^3^, the two groups were treated with indicated treatments (the vehicle group, and oridonin group with 10 mg/kg), respectively. Then, the diameter of tumors and body weight of nude mice were measured every 3 days, and the tumor volume was calculated as 1/2 × (length) × (width)^2^. Ultimately, the nude mice were sacrificed to harvest the tumors at day 18 and xenografts were used for the subsequent western blotting assay.

### Statistical analysis

Data were represented as mean ± standard deviation*.* All statistical analyses were performed using GraphPad Prism (vesion 6.0) software. Statistical differences in different groups were compared by Student's *t*-test (two-sided) or one-way analysis of variance (ANOVA). A value of *P <* 0.05 was considered statistically significant.

## Results

### Oridonin inhibited the proliferation of thyroid cancer cells

**Figure 1:**
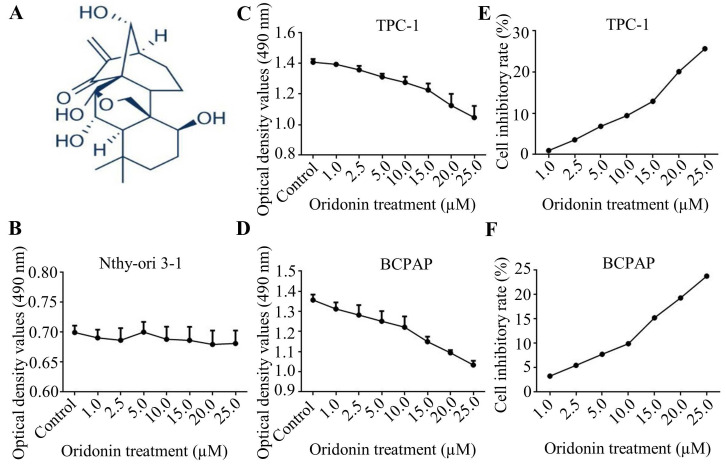
Oridonin suppressed the proliferation of thyroid cancer cells. (A) The chemical structure of oridonin. Nthy-ori 3-1 (B), TPC-1 (C, E) and BCPAP (D, F) cells with 90 percent density were treated with different concentrations of oridonin for 24 h (0, 1, 2.5, 5, 10, 15, 20, 25 μM). Then the viability of normal thyroid epithelial Nthy-ori 3-1 cell line and thyroid cancer TPC-1 and BCPAP cell lines were evaluated by MTT assays. The optical density and cell inhibitory rate were presented. Error bars indicated mean ± SD. Three independent experiments were performed.

Firstly, the molecular structure of oridonin was presented in Fig.** 1A**. Since oridonin gained a great attention in recent years with the beneficial anticancer potential, we initially detected the cytotoxic effect of oridonin in human thyroid epithelial Nthy-ori 3-1 cell and thyroid cancer TPC-1 and BCPAP cell lines with a modified MTT assay. The results showed that oridonin had no remarkable cytotoxic effect on thyroid epithelial cell, which was consistent with the previous results 13. On the other hand, oridonin presented anti-proliferative effect on thyroid cancer TPC-1 and BCPAP cells in a concentration-dependent manner (Fig. **1B**-**1D**). In addition, we found that oridonin exhibited no significant cytotoxicity, with an inhibitory rate of less than 10%, on the two thyroid cancer cell lines at 24 h when the concentration of oridonin was no higher than 10 μM. Therefore, a concentration of oridonin no higher than 10 μM (0, 2.5, 5.0, 10.0 μM) at 24 h was used in subsequent experiments, to exclude the interference of cell proliferation.

### Oridonin inhibited the migration and invasion of human thyroid cancer cells

**Figure 2:**
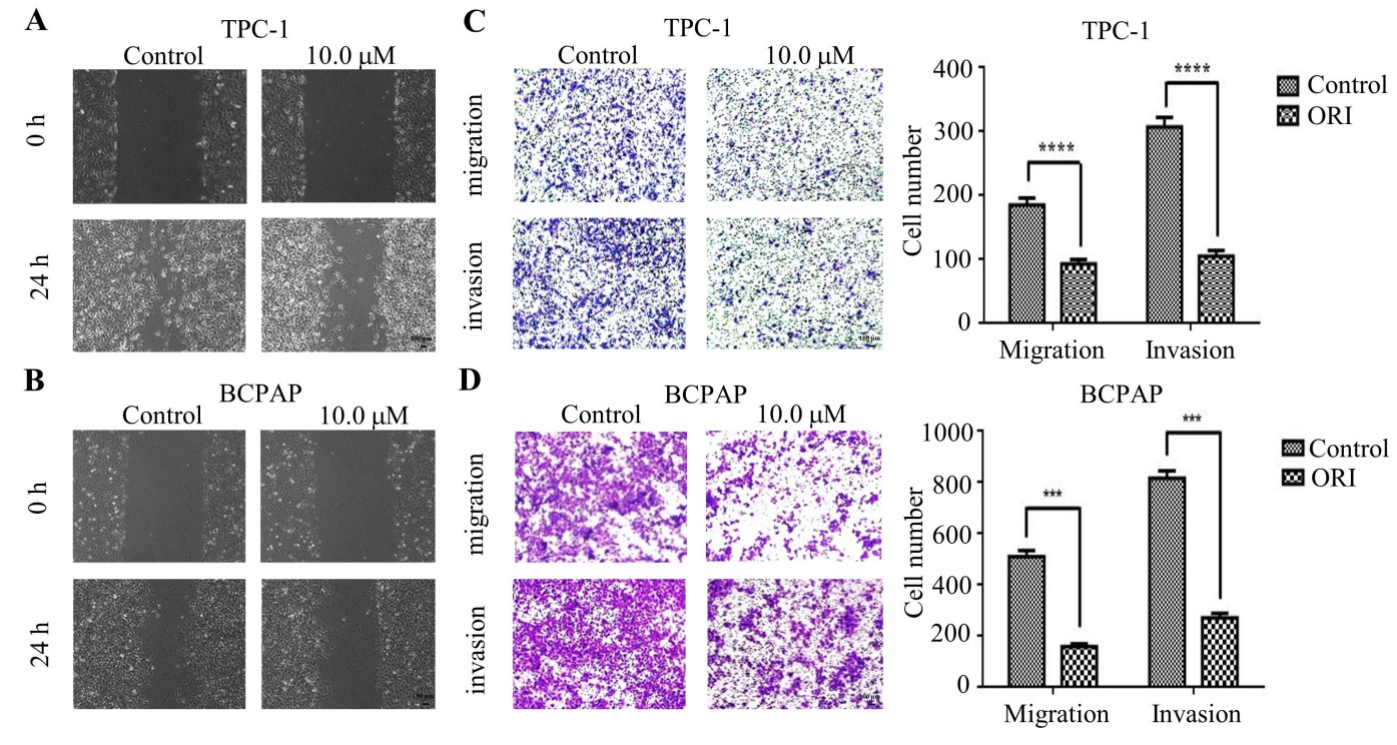
Oridonin inhibited the migratory and invasive activity of thyroid cancer TPC-1 and BCPAP cells. The scratch width of thyroid cancer TPC-1 (A) and BCPAP (B) cell lines was evaluated at the presence or absence of oridonin. Meanwhile, the number of migrated or invaded cells (C, D) per chamber was counted upon DMSO or oridonin treatment, as evidenced by transwell migration assay and Matrigel invasion assay (magnification, ×100)(***P < 0.001, ****P<0.0001). Three independent experiments were performed.

To identify the role of oridnoin in migratory and invasive property in thyroid cancer cells, wound healing assay was conducted to verify the anti-metastatic effect of oridonin. The results demonstrated that the scratch width of oridonin group was much wider than that in control group in both thyroid cancer TPC-1 and BCPAP cell lines (Fig. **2A** and **2B**). To further uncover the impact of oridonin on metastasis of thyroid cancer cells, the effects of oridonin on cell migration and invasion were assessed by transwell migration assay and matrigel invasion assay. As shown in Fig. **2C**, the migratory ability of TPC-1 cells was weakened by oridonin treatment. The similar results were also conducted in thyroid cancer BCPAP cells under oridonin treatment (Fig. **2D**). For thyroid cancer cell invasion, oridonin could remarkably suppress cell invasion in thyroid cancer TPC-1 and BCPAP cell lines (Fig.** 2C** and **2D**).

In summary, the results indicated that oridonin exerted a strong anti-metastatic ability in human thyroid cancer cells.

### Oridonin regulated expressions of epithelial-mesenchymal transition-related indicators in thyroid cancer cells

**Figure 3:**
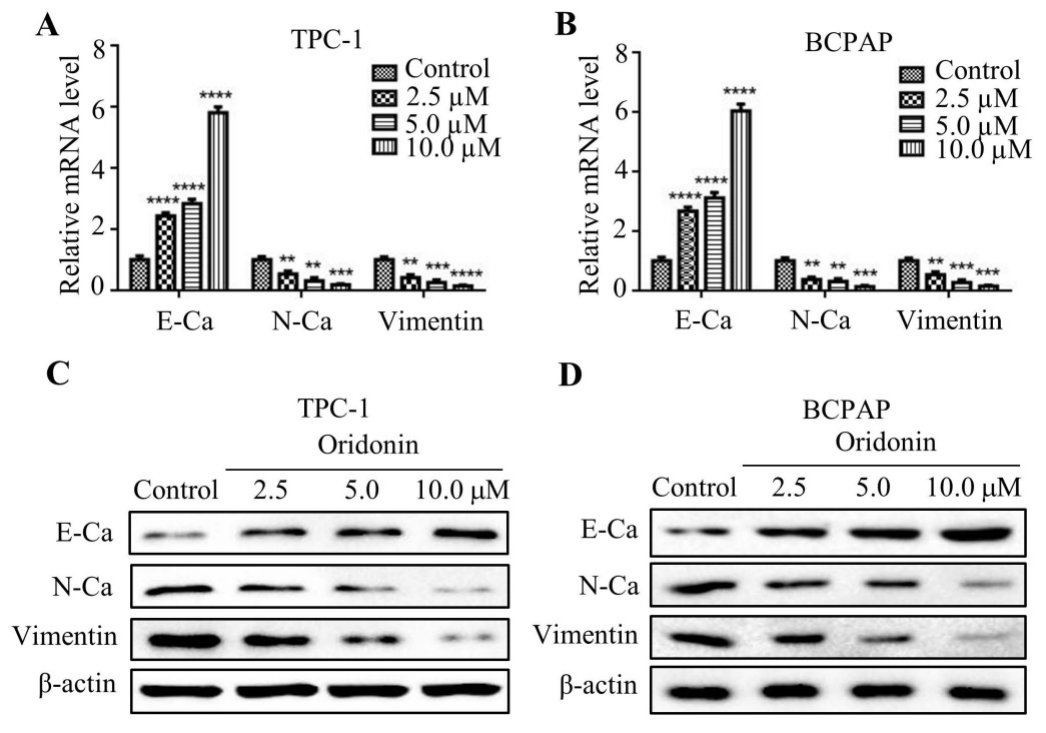
Oridonin inhibited the mRNA and protein levels of epithelial-mesenchymal transition markers in thyroid cancer cells. The mRNA levels of E-Cadherin, N-Cadherin and Vimentin in TPC-1 (A) and BCPAP (B) cells were detected by quantitative real-time PCR (**P*<* 0.01, ***P<0.001, ****P<0.0001). Three independent experiments were performed. TPC-1 (C) and BCPAP (D) cells treated with DMSO or oridonin were subjected to western blotting for E-Ca, N-Ca, Vimentin and β-actin.

Numerous studies reported that epithelial-mesenchymal transition is associated with cancer migration and invasion. In consideration of that, the mRNA expression of E-cadherin, N-cadherin and Vimentin were detected by quantitative real-time PCR. As shown in Fig. **3A** and** 3B**, oridonin was found to effectively upregulate E-cadherin mRNA level, while to downregulate N-cadherin and Vimentin mRNA levels in a concentration-dependent manner. Consistent with those results, oridonin dose-dependently decreased N-cadherin and Vimentin protein levels, while increased E-cadherin protein level (Fig. **3C** and **3D**). These findings revealed that oridonin could modulate EMT in thyroid cancer cells.

### Oridonin inhibited epithelial-mesenchymal transition of thyroid cancer cells by downregulating JAK2-STAT3 pathway

**Figure 4:**
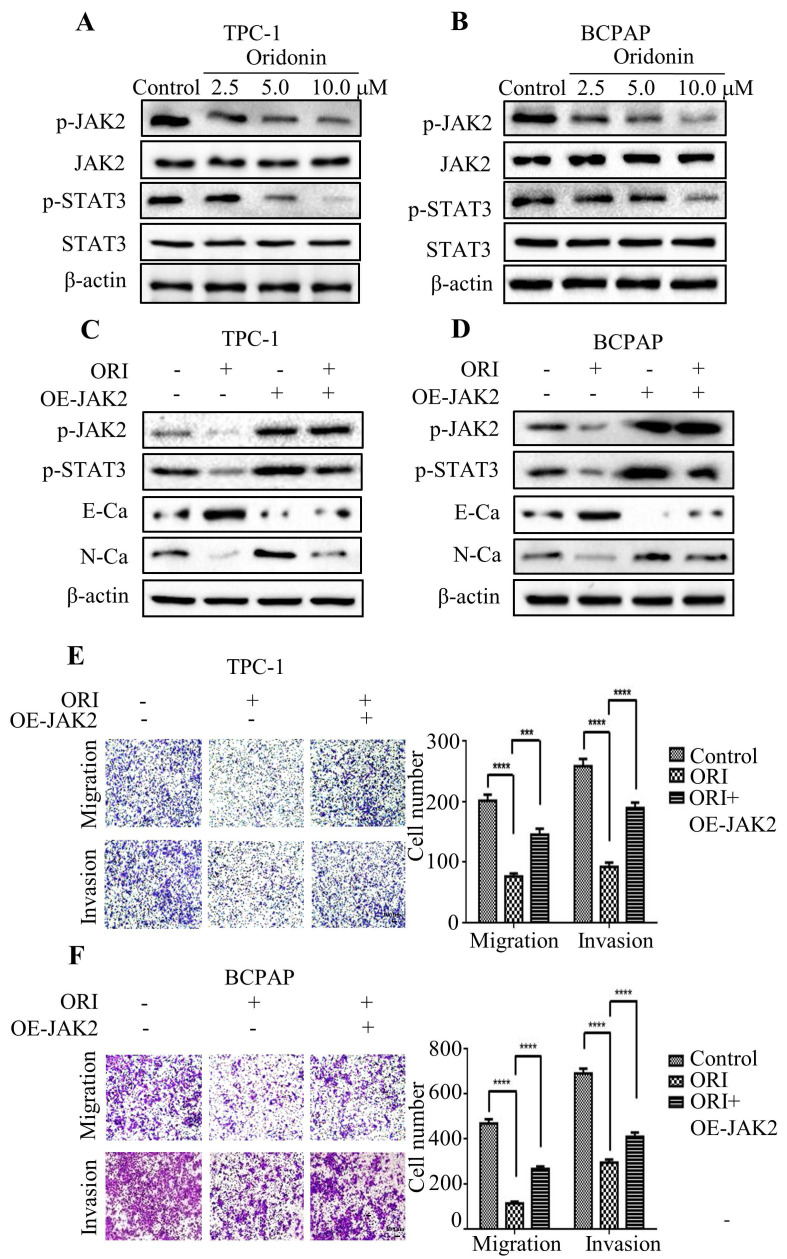
Overexpression of JAK2 impaired the anti-metastatic effect of oridonin in thyroid cancer cells. Western blotting was used to detect the protein levels of phosphorylated-JAK2, JAK2, phosphorylated-STAT3, STAT3, E-Ca, N-Ca and β-actin in thyroid cancer TPC-1 and BCPAP cells upon oridonin treatment (A, B) or the co-treatment of oridonin and JAK2 overexpression (C, D). Transwell migration assay and Matrigel invasion assay were conducted in TPC-1 (E) and BCPAP (F) cells with the combination treatment of oridonin and JAK2 overexpression. The fields were randomly chosen and visualized by microscopy (magnification, ×100) (***P<0.001, ****P<0.0001). Three independent experiments were performed.

**Figure 5:**
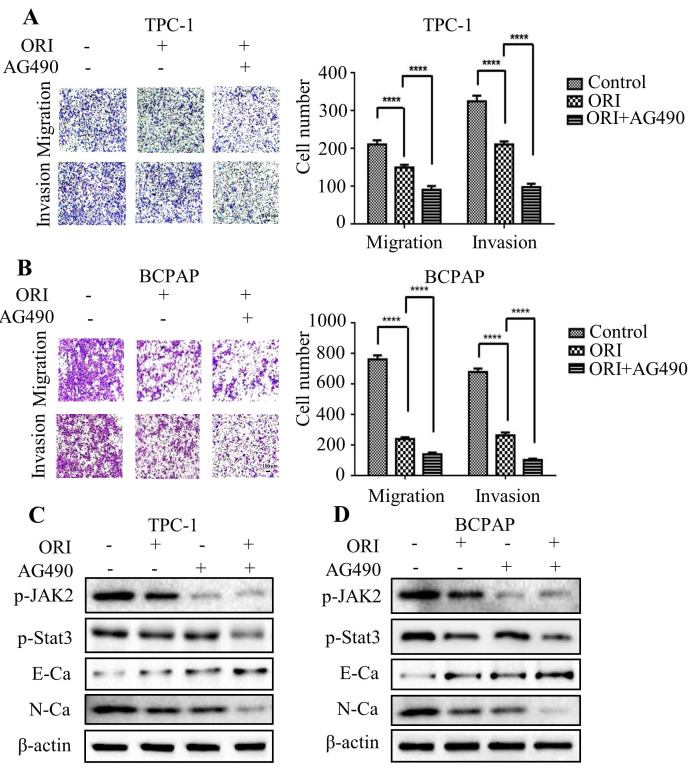
AG490 strengthened the anti-migratory and anti-invasive characteristic of oridonin in thyroid cancer cells. Transwell migration assay and Matrigel invasion assay were performed in TPC-1 (A) and BCPAP (B) cells with the combination treatment of oridonin and AG490. The fields were randomly chosen and visualized by microscopy (magnification, ×100) (****P<0.0001). Three independent experiments were performed. Western blotting was used to detect the protein levels of phosphorylated-JAK2, phosphorylated-STAT3, E-Ca, N-Ca and β-actin in thyroid cancer TPC-1 (C) and BCPAP (D) cells upon co-treatment of oridonin and AG490.

Accumulating evidence indicated that JAK2/STAT3 was closely correlated with cancer metastasis and progression 14-15. To investigate the regulation of cell migration and invasion of thyroid cancer cells by oridonin, western blotting analysis was applied to detect the protein levels of p-JAK2, JAK2, p-STAT3 and STAT3. Interestingly, oridonin effectively repressed p-JAK2 and p-STAT3 protein levels in thyroid cancer TPC-1 and BCPAP cell lines in a concentration-dependent manner (Fig. **4A** and **4B**). To further confirm whether JAK2/STAT3 pathway is involved with anti-metastatic characteristic of oridonin in human thyroid cancer cells or not, we overexpressed JAK2 by transiently transfecting JAK2 plasmid. We found that overexpression of JAK2 could partially reverse the change of p-STAT3, E-cadherin and N-cadherin protein levels induced by oridonin in thyroid cancer TPC-1 and BCPAP cells (Fig. **4C** and **4D**). In addition, overexpressing JAK2 could weaken the anti-metastatic capacity of oridonin in thyroid cancer (Fig. **4E** and **4F**). Then AG490 was introduced as an inhibitor role to antagonize JAK2. In contrast to the results of JAK2 overexpression, our findings revealed that AG490 could further strengthen the inhibitory effect of oridonin on thyroid cancer migration and invasion (Fig.** 5A** and** 5B**). Furthermore, the change of p-STAT3, E-cadherin and N-cadherin protein levels regulated by oridonin could be enhanced by AG490 (Fig.** 5C** and** 5D**). These findings strongly suggested that oridonin exerted anti-metastatic effect via the inactivation of the JAK2/STAT3 signaling pathway.

### Oridonin inhibited angiogenesis in thyroid cancer cells via downregulating JAK2-STAT3 pathway

**Figure 6:**
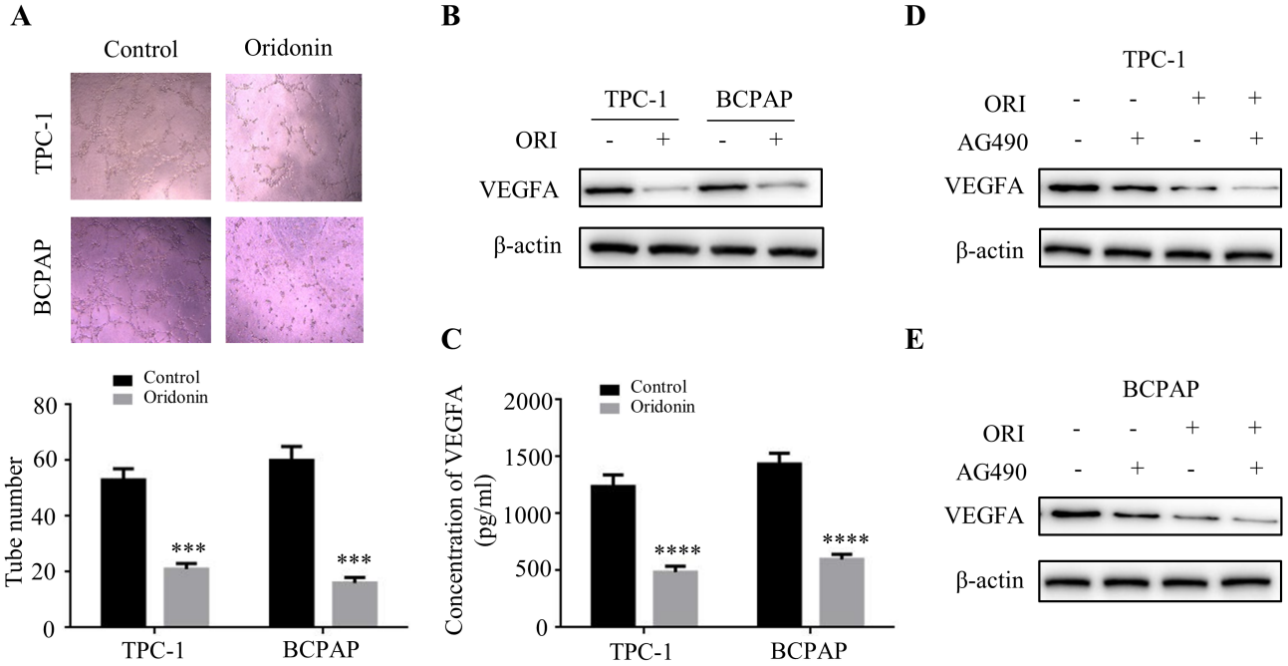
Oridonin inhibited angiogenesis of thyroid cancer via JAK2/STAT3 signaling. (A) Tube formation assay was used to evaluate the formation of new vessels. The representative pictures and quantification analysis of tube number of HUVECs treated with serum-free medium (SFM) or conditional mediums (CMs) were shown (magnification, ×100) (***P < 0.001). The conditional mediums were obtained from thyroid cancer TPC-1 and BCPAP cells upon oridonin treatment. (B) Western blotting was used to detect the protein levels of VEGFA and β-actin in thyroid cancer TPC-1 and BCPAP cells exposed to oridonin or not. (C) The concentration of VEGFA in conditional mediums (CMs) was detected by ELISA assay (magnification, ×100) (****P < 0.0001). CMs were collected from TPC-1 and BCPAP cells upon oridonin treatment. (D) The protein levels of VEGFA and β-actin in TPC-1 and BCPAP cells upon co-treatment of oridonin and AG490 were shown by western blotting.

The angiogenesis is critical for tumor metastasis and progression 16. To explore the impact of oridonin on the formation of new vessels, the tube formation assay was performed using HUVECs. The findings revealed that conditional medium exposed to oridonin could impair the formation of tube-like structure in matrigel in thyroid cancer TPC-1 and BCPAP cells (Fig. **6A**). The results indicated that oridonin played a suppressor role in thyroid cancer angiogenesis. Accumulating evidence showed that VEGFA is correlated with the formation of new vessels 17. Indeed, we found that VEGFA protein level was downregulated upon oridonin treatment in these two cell lines (Fig. **6B**). Meanwhile, the results of ELISA assay demonstrated that concentration of VEGFA decreased in CMs under oridonin treatment (Fig. **6C**). These data suggested that oridonin regulated VEGFA expression to repress thyroid cancer angiogenesis. To further investigate the mechanism of oridonin on regulating VEGFA in thyroid cancer cells, the cells were co-treated with oridonin and JAK2 inhibitor, AG490. As expected, the data showed that AG490 could further weaken the protein level of VEGFA upon oridonin treatment in TPC-1 and BCPAP cells (Fig. **6D** and **6E**). Taken together, these data strongly suggested that oridonin could suppress VEGFA expression via JAK2/STAT3 pathway in thyroid cancer cells.

### Oridonin inhibited tumorigenicity and angiogenesis *in vivo*

**Figure 7:**
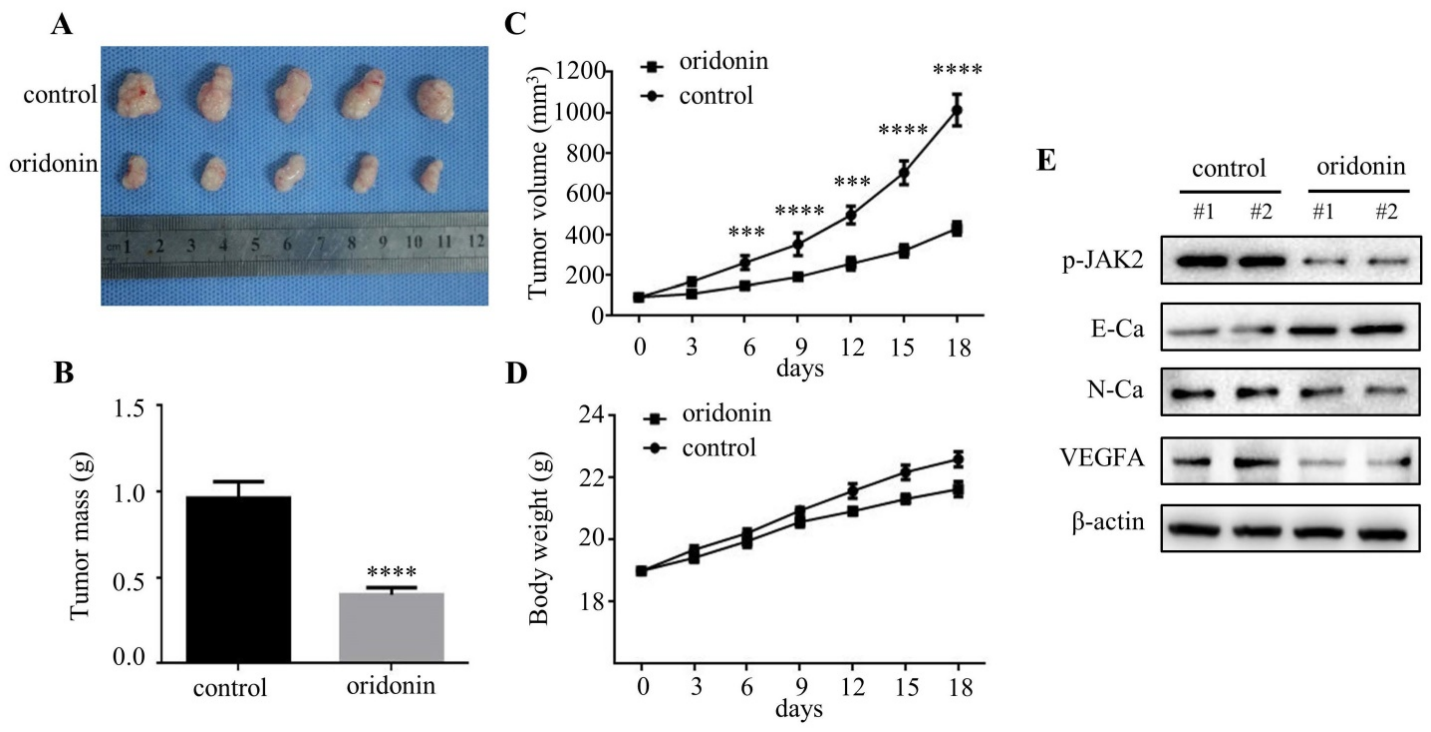
Oridonin significantly decreased thyroid cancer tumorigenicity and angiogenesis in a nude mouse xenograft model. (A) Representative pictures and quantification analysis of xenografts in control and oridonin groups were presented in 18 days. 5×10^6^ TPC-1 cells were subcutaneously injected into the right flank of nude mice. (B) The quantification analysis of tumor mass in control and oridonin groups were shown as mean ± SD of five mice (****P < 0.0001). (C) Tumor volumes and (D) body weight of nude mice were detected every 3 days. The statistical results were presented as mean ± SD of five mice (***P < 0.001, ****P < 0.0001). (E) The protein levels of phosphorylated JAK2, E-cadherin, N-cadherin, VEGFA and β-actin were detected by Western blot. The proteins were collected from the dissected tumor tissues of control and oridonin groups).

To further clarify the therapeutic effect of oridonin in thyroid cancer *in vivo*, we established a nude mice xenograft using TPC-1 cells. The results revealed that the tumor mass and volume in oridonin group were dramatically decreased compared with that in control group (Figure.**7A-7C**), while the body weights were similar in these two groups (Figure **7D**). Subsequently, the data of western blotting showed that the protein levels of phosphorylated-JAK2, N-cadherin and VEGFA were reduced in tumor tissues exposed to oridonin, while E-cadherin protein level was elevated (Figure **7E**). These findings indicated that oridonin could inhibit thyroid cancer tumorigenesis *in vivo*.

## Discussion

In recent years, oridonin gained great attention due to its strong anticancer activity against a variety of tumors 18-19. Accumulating studies showed that oridonin is associated with migration, invasion, EMT and angiogenesis of cancer. It was reported that oridonin could suppress metastasis of ovarian cancer via the inhibition of mTOR pathway 8. Oridonin was also found to negatively regulate Wnt/β-catenin signaling pathway to repress migration and EMT of pancreatic cancer cells 20. Moreover, oridonin could inhibit colon cancer angiogenesis and induce vessel normalization 21. In the present study, we found that oridonin could effectively repress the migratory and invasive abilities of thyroid cancer TPC-1 and BCPAP cells at a low concentration (less than 10 μM), which had no significant inhibition on cell viability.

EMT is a complicated process that was related to the loss of epithelial characteristics and cell polarity, and the acquisition of mesenchymal properties 22. Increasing evidence have showed that EMT is closely correlated with cancer development and metastasis 23. Studies showed that oridonin could inhibit EMT of small cell lung cancer cells by downregulating FAK (Focal Adhesion Kinase)-ERK (extracellular regulated protein kinases) 1/2 signaling pathway 24. Moreover, it was reported that oridonin inhibited EMT of pancreatic cancer cells by inactivation of lncRNA AFAP1-AS1 25. The findings of the present study revealed that the mRNA and protein levels of E-cadherin were significantly increased upon oridonin treatment, while the mRNA and protein levels of N-cadherin and vimentin were sharply decreased by oridonin, indicating that oridonin could partially impede EMT of thyroid cancer TPC-1 and BCPAP cell lines.

To further explore the underlying mechanism of oridonin on anti-metastatic activity, many signaling pathways were involved with the process, which include TGF (Transforming Growth Factor)-β1/Smad2/3 signaling and PI3K/Akt/GSK-3β (glycogen synthase kinase-3β) signaling 26-27. Studies also revealed that FAK-ERK1/2 signaling pathway was involved with the antagonism of EMT by oridonin in small cell lung cancer cells 24. For JAK2/STAT3 signaling pathway, it was reported to plays vital roles in the EMT, metastasis and drug resistance of various cancers 28-29. Studies showed that miR-630 may inhibit metastasis and EMT of papillary thyroid cancer via the inactivation of JAK2/STAT3 signaling pathway 30. Additionally, REX1 could promote EMT-induced cell metastasis by the activation of JAK2/STAT3 signaling pathway 31. Thus, we hypothesized JAK2/STAT3 signaling pathway participated in the anti-migratory and anti-invasive effects of oridonin on thyroid cancer cells. The current study exhibited an effective decrease of p-JAK2 and p-STAT3 protein levels upon oridonin treatment in thyroid cancer TPC-1 and BCPAP cells, as evidenced by western blotting. To further validate its role in oridonin-regulated EMT in thyroid cancer, JAK2 overexpression plasmid was used to overexpress JAK2. The subsequent investigations demonstrated that overexpression of JAK2 weakened the anti-metastatic effect of oridonin and partially attenuated MET (mesenchymal-to-epithelial transition) by oridonin. In addition, AG490, a JAK2 antagonist, enhanced the anti-migratory and anti-invasive effects of oridonin on thyroid cancer cells, which were confirmed by western blotting, Transwell migration assay and Matrigel invasion assay. These results suggested that the anti-metastatic characteristic of oridonin may be mediated by JAK2/STAT3 signaling pathway.

For the angiogenesis, it is widely reported that angiogenesis is crucial for the tumor progression. Consistent with the previous studies, the results of the present study revealed that oridonin suppressed angiogenesis of thyroid cancer. Studies showed that oridonin could inhibit VEGFA-associated angiogenesis of breast cancer 32. In addition, it has been reported that oridonin inhibited VEGF-induced VEGFR-2 signaling to play an anti-angiogenic role in human umbilical vein endothelial 33. In our study, the findings demonstrated that oridonin could modulate the protein level of VEGFA to repress thyroid cancer angiogenesis, as evidenced by the western blotting assay and ELISA assay. Meanwhile, the results of western blotting revealed that oridonin inhibited the angiogenesis of thyroid cancer via JAK2 signaling. These data strongly suggested the anti-angiogenic effect of oridonin in thyroid cancer via JAK2-STAT3 signaling. In accordance with the results *in vitro*, the *in vivo* results showed that oridonin significantly inhibited tumorigenicity and exerted anti-angiogenic property in thyroid cancer xenografts.

## Conclusion

Our studies revealed that oridonin inhibited metastatic phenotype, angiogenesis and modulated EMT in thyroid cancer *in vitro* and *vivo* through downregulation of JAK2/STAT3 signaling pathway. In addition, the findings indicated that JAK2 may be a potential therapeutic target of oridonin against thyroid cancer.
